# Effects of Pure and Mixed Autochthonous *Torulaspora delbrueckii* and *Saccharomyces cerevisiae* on Fermentation and Volatile Compounds of Narince Wines

**DOI:** 10.3390/foods7090147

**Published:** 2018-09-05

**Authors:** Ebru Arslan, Zeynep Dilan Çelik, Turgut Cabaroğlu

**Affiliations:** 1 Department of Food Engineering, Faculty of Agriculture, Çukurova University, 01330 Adana, Turkey; ebru.arslann02@gmail.com (E.A.); z.d.celik@gmail.com (Z.D.Ç.); 2 Department of Biotechnology, Institute of Natural and Applied Science, Cukurova University, 01330 Adana, Turkey

**Keywords:** *Saccharomyces cerevisiae*, *Torulaspora delbrueckii*, Narince, autochthonous, mixed culture

## Abstract

The cultivar of Narince is a native white grape variety of *Vitis vinifera*, grown in Tokat city, the Mid-Black Sea Region of Anatolia. In this study, the effects of pure and mixed autochthonous *Torulaspora delbrueckii*-214 and *Saccharomyces cerevisiae*-1088 cultures on the fermentation behavior and aroma compounds of Narince wines were investigated. Volatile compounds formed in wines were extracted using a liquid–liquid extraction method and determined by GC-MS-FID. Narince grape must was fermented in duplicate, under the following three conditions. Two pure cultures of *T. delbrueckii*-214 and *S. cerevisiae*-1088 and a mixture of *T. delbrueckii*-214 and *S. cerevisiae*-1088 (1:1). The presence of the non-*Saccharomyces T. delbrueckii*-214 yeast slowed down the fermentation and produced a lower level of ethanol and a higher levels of glycerol and volatile acid. Only the pure culture of *T. delbrueckii*-214 was unable to finish fermentation. On the other hand, mixed culture fermentation improved the aroma intensity and complexity of wine due to increased levels of higher alcohols and esters. According to sensory analysis, wine fermented with mixed culture was the most preferred wine followed by wine inoculated with pure *S. cerevisiae*-1088. This study confirms the role of *T. delbrueckii* in wine aroma and the potential of non-*Saccharomyces* use in winemaking.

## 1. Introduction

The fermentation of grape juice into wine is a complex microbial process carried out by yeasts belonging to different species of *Saccharomyces* and non-*Saccharomyces* yeasts [1,2,3]. In modern winemaking, alcoholic fermentation is typically carried out using selected strains (microbial starter) of *S. cerevisiae*. However, during traditional fermentation of the grape must different yeast species/strains that are naturally present on the berries, grapes, or in the winery environment are involved into the fermentation [4]. The early stages of natural fermentation are dominated by non-*Saccharomyces* yeasts from the genera, *Candida*, *Hanseniaspora*, *Torulaspora*, and *Pichia*. These yeasts have low fermentation activity but compose multiple flavor compounds such as, esters, higher alcohols, terpenes, and glycerol [5]. After the first days of fermentation, strongly fermenting and ethanol tolerant species of *S. cerevisiae* become dominant and complete the wine fermentation [6,7]. Generally, non-*Saccharomyces* species are considered to be of secondary significance or undesirable during alcoholic fermentation. However, several authors have reported that the dominance of non-*Saccharomyces* yeasts during the early stages of fermentation have a major impact on the organoleptic quality of wines [8,9,10]. Also, mixed culture fermentations of several non-*Saccharomyces* species, such as, *Hanseniaspora uvarum*, *T. delbrueckii*, *Kluyveromyces thermotolerans*, and *Pichia kluyveri*, together with *S. cerevisiae* have been investigated during winemaking. The positive impact with mixed culture fermentation protocols stimulated the investigation and selection of new non-*Saccharomyces* starters able to carry out alcoholic fermentation together with *S. cerevisiae* [9,11,12]. *T. delbrueckii* is one of the non-*Saccharomyces* yeast species which is commercialized because of its positive impact on the organoleptic properties of wines [11]. However the use of *T. delbrueckii* pure culture leads to ‘stuck’ fermentation. It has been reported that mixed culture of selected strains of this species with *S. cerevisiae* modulate wine flavor and provide complete alcoholic fermentation [1]. Several authors have indicated that besides the reduction of off-flavors, compounds like acetaldehyde, and acetoin, the mixed inoculation of these two yeasts species gives a steady increase in 2-phenylethanol, terpenes, and lactones [1,11,12,13].

The yeast influence on wine aroma is not only species dependent but also strain dependent [4]. In recent years, there is increasing interest among wine-researchers and winemakers to select local strains with the aim to select starter cultures that are potentially well-adapted to a specific grape must reflecting the biodiversity of a given region, which supports the notion that specific autochthonous yeast strains can be associated with a terroir. Many studies state that production of wines by autochthonous yeasts isolated from its *terroir can* contribute to the regional character of the wine, especially the wine flavor [14,15,16]. In the present study, autochthonous *S. cerevisiae* and *T. delbrueckii* yeasts previously isolated from the spontaneous fermentation of Narince grapes were used.

Cv. Narince is the native, and commercially important, unique white wine grape variety of the Tokat region of Turkey. Narince vineyards of the Tokat region have remarkable historical value dating back to the Hittite times—civilized 4000 years ago. Narince makes straw yellow colored wines with floral notes, yellow fruit, and citrus aromas on the nose. On the palate, it produces round, medium to full bodied wines, balanced with good acidity.

The aim of this study was to investigate the effects on the fermentation and volatile composition of Narince wines fermented by pure and mixed autochthonous *T. delbrueckii*-214 and *S. cerevisiae*-1088 cultures. To our knowledge this is the first study about the effects of autochthonous pure and mixed cultures of *S. cerevisiae* and *T. delbrueckii* yeasts on the volatile composition of Narince wines.

## 2. Material and Methods

### 2.1. Yeast Strains 

The *T. delbrueckii*-214 (Accession number: MG017548) and *S. cerevisiae*-1088 (Accession number: MG017577) yeasts culture used in this study were previously isolated from spontaneous fermentations of Narince grapes. These strains were chosen due to their good technological properties such as fermentation power, fermentation vigor, higher alcohol production, and low production of volatile acidity, ethyl acetate, and acetaldehyde. The fermentation rate of *T. delbrueckii*-214 was 0.41 CO_2_ g/L·h, the fermentation power was 9. 40% *v*/*v* ethanol, and it produced 0.78 g/L volatile acidity, 6.05 g/L glycerol, 200 mg/L higher alcohol, 34 mg/L ethyl acetate, and 13 mg/L acetaldehyde. The technological properties of *S. cerevisiae*-1088 were previously reported by Çelik et al. [17].

### 2.2. Culture Media and Chemicals

Yeast peptone dextrose agar (YPD), YPD broth, and L-lysine agar were purchased from Sigma Aldrich (St Louis, MO, USA). Dichloromethane (≥99.9% purity), sodium sulfate anhydrous (99%), internal standard (4-nonanol), and a mixture of n-alkane standards ranging from C_8_–C_40_ were purchased from Merck (Darmstadt, Germany). Standard volatile compounds used in the study were obtained from Sigma Aldrich (St Louis, MO, USA).

### 2.3. Fermentations

Grapes from *Vitis vinifera* L. Narince were harvested during the 2016 vintage in the commercial vineyard of Kavaklıdere, and fermentations were conducted at the experimental winery of the University of Çukurova. Narince grapes were at optimum maturity with, 20.4 brix, 5.44 g/L titratable acids as tartaric acid, and a pH of 3.60. Grapes were crushed and 50 mg/L of SO_2_ was added. After pressing, the juice was allowed to settle (10 °C for 12 h), separated from the lees and randomly distributed into 20 L glass fermentation bottles. Three different fermentations were conducted: two with the pure culture and one with the mixed culture. *T. delbrueckii*-2014 and *S. cerevisae*-1088 were inoculated at the same time (1:1) for mixed culture fermentation [11]. All fermentations were carried out in duplicate using the standard protocol for white wines: Yeast culture of *T. delbrueckii*-2014 and *S. cerevisiae*-1088 were previously grown in YPD broth at 28 °C for 24 h; following this, the cells were recovered by centrifugation, washed with sterile water, and added to the must at 10^6^ cells mL/L. All fermentations were conducted at 18 °C in a cold room. The normal development of alcoholic fermentation was checked by daily monitoring of the temperature and density values. During the first day after the initiation of fermentation, in the middle (which contained % 50 sugar), and at the end of fermentation, samples of must and wine, diluted in 0.1% peptone-water (decimal dilutions), were inoculated onto plates of YPD, Lysine, and modified YPD agar (% 10 ethanol, *v*/*v* and 2 g/L potassium metabisulfite) supplemented with chloramphenicol and sodium propionate to inhibit bacteria and filamentous fungi, respectively. Plates with YPD and modified YPD medium were incubated at 28 °C for 2 days before counting, whereas plates with lysine agar were incubated at 25 °C for 3–5 days before counting [11,18,19]. At the end of the alcoholic fermentation, all wines were racked off gross lees and sulfur dioxide was added to yield a free SO_2_ of 50 mg/L. After this, the wines were bottled and stored at 13–18 °C until analysis.

### 2.4. Chemical Analysis of Wines

Density, alcohol, total acidity, pH, volatile acidity, reducing sugar, dry matter, and total SO_2_, were measured according to the methods outlined by OIV [19]; glucose, fructose, and glycerol were quantified using HPLC (Shimadzu, Kyoto, Japan) according to OIV and Erten [20,21].

### 2.5. Volatile Compounds Analysis

A 100 mL wine sample was transferred into a 500 mL Erlenmeyer flask and cooled to 0 °C in an ice bath under nitrogen; 40 micrograms of 4-nonanol was added as an internal standard because of its high recovery [22]. Dichloromethane (40 mL) was added and the mixture was stirred at 700 rpm for 30 min. Then the mixture was centrifuged at 4 °C (9000× *g*, 15 min). The organic phase was recovered. The organic extract dried over sodium sulfate and concentrated to a volume of 1 mL with a Vigreux distillation column prior to gas chromatography/mass spectrometry (GC/MS) analysis. Each sample was extracted in triplicate and the concentration of volatiles, as 4-nonanol equivalent, was obtained as the mean of three repetitions [22].

### 2.6. GC-MS-FID Conditions and Identification of Volatile Compounds

An Agilent 6890 gas chromatograph (GC) was equipped with a flame ionization detector (FID) (Wilmington, DE, USA) and an Agilent 5973-Network mass selective detector (MSD). Volatile compounds were separated on DB-Wax (30 m length × 0.25 mm i.d. × 0.5 µm thickness; J&W Scientific Folsom, CA, USA) column and 3 µL sample of extract was injected. Injector and FID detectors were set at 250 °C. The flow rate of carrier gas (helium) was 1.5 mL/min. The oven temperature of the DB-Wax column was increased from 40 °C (after 3 min holding) to 90 °C at a rate of 2 °C/min, then at a rate of 3 °C/min to 130 °C and at a rate of 4 °C/min to 240 °C with a final hold, at 240 °C for 12 min. The same oven temperature programs were used for the mass selective detector. MS scan conditions: source temperature 120 °C, interface temperature 250 °C, EI energy 70 eV, mass scan range 29–350 a.m.u., and a scan rate of 1.0 scan/s [22,23,24]. The identification of compounds was made up of the following parameters: commercial spectra database (Wiley 6, NIST 98), retention indices, an internal library created from the previous laboratory studies. Some of the identifications were confirmed by the injection of the chemical standards into the GC-MS system. Retention indices of the compounds were calculated by using an n-alkane series [22,23,24].

The determination of acetaldehyde and ethyl acetate was carried out by direct injection of 1 μL samples into a gas chromatograph, Agilent 6890 N equipped with FID. Acetaldehyde and ethyl acetate separated using a Chrompack CP-WAX-57CB capillary column (0.25 mm i.d. × 60 m × 0.4 μm film thickness) (Middelburg, The Netherlands). GC settings: injection temperature: 160 °C; oven temperature: 5 min at 40 °C, then increased by 4 °C per minute up to 102 °C and 2 °C per minute up to 125 °C and hold for 5 min, then 3 °C per minute up to 160 °C, 6 °C per minute up to 200, and finally hold 5 min at 200 °C; carrier gas: He (1.3 mL/min); split rate: 1:50. The quantification was performed by using equation stated reference of Cabaroğlu et al. [25] with internal standard (4-methyl-2-pentanol) method. Analysis was done in triplicate.

### 2.7. Sensory Analysis

The sensory characteristics of the final wines were evaluated according to Lawless and Heymann [26]. The sensory panel was comprised of 6 females and 4 males, 25–50 years of age, all belonging to the laboratory staff and having substantial experience of sensory analysis. The panelist used a 10 cm scale, from 0 (no defect) to 10 (very strong defect perceptible). Each panelist smelled and then tasted the wines in the tasting glass to detect the intensity of the 13 attributes (herb tea, citrus, tropical fruit, stone fruit, honey, herbaceous, cooked vegetable, complexity, acidity, after taste, aroma, and general impression). Sensory analysis was done in five-booth sensory panel room at 22 °C equipped with white fluorescent lighting. Wines were served (50 mL at 10 °C) in tulip-shaped wine glasses covered by glass petri dishes. The tasting glasses were coded with different three-digit numbers.

### 2.8. Statistical Analysis

The results were compared by the analysis of variance (ANOVA) using SPSS (for Windows version 20.0). Duncan’s multiple-range tests were used to compare the significant differences of the mean values with *p* < 0.05.

## 3. Results and Discussion

### 3.1. Growth of the T. delbrueckii-2014 and S. cerevisiae-1088 Populations During Fermentation

**Figure 1:**
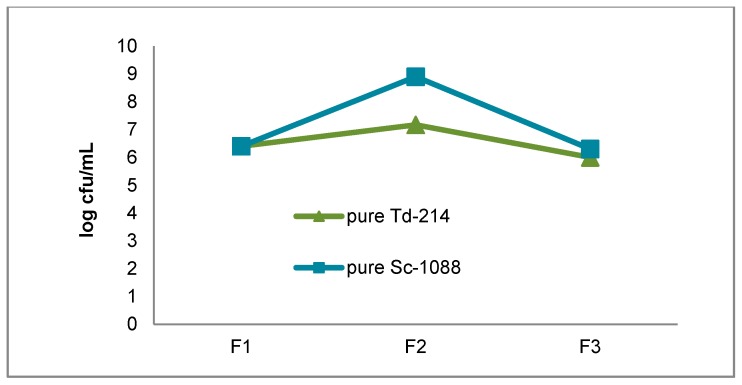
The growth of yeasts during pure culture fermentations. Pure Td-214: pure *T. delbrueckii*-214, pure Sc-1088: pure *S. cerevisiae*-1088. F1: beginning of fermentation. F2: middle of fermentation. F3: end of fermentation.

**Figure 2:**
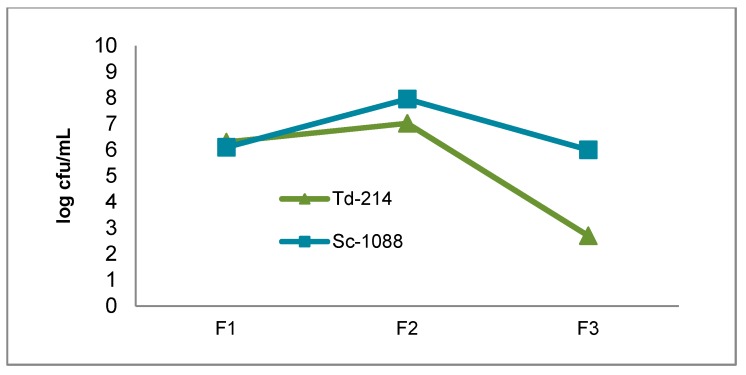
The growth of yeasts during mixed culture fermentation. Td-214: *T. delbrueckii*-214, Sc-1088: *S. cerevisiae*-1088. F1: beginning of fermentation. F2: middle of fermentation. F3: end of fermentation.

The viable yeasts population in pure and mixed culture fermentations are shown in Figure 1 and Figure 2. In all fermentations the must was inoculated with 5 × 10^6^ viable cells/mL for both *T. delbrueckii*-214 and *S. cerevisiae*-1088 and the initial must yeasts concentration following inoculation ranged from 6.1 to 6.4 log cfu/mL. The pure culture of *T. delbrueckii*-214 and *S. cerevisiae*-1088 populations reached a maximum size of 7.1 to 8.9 log cfu/mL at the middle of fermentation. At the end of fermentation both pure *T. delbrueckii*-214 and *S. cerevisiae*-1088 populations slowly decreased, as was expected. In the mixed culture fermentation both species were added at the same time. The maximum population reached during alcoholic fermentation by *T. delbrueckii*-214 and *S. cerevisiae*-1088 was higher when inoculated alone rather than in mixed culture. At the end of the mixed culture fermentation the population of the viable *T. delbruecki*-214 culture had decreased. Renault et al. [27] have reported that, while *S. cerevisiae* yeast is able to quickly grow under strictly anaerobic conditions, *T. delbrueckii* is affected by a deficiency of oxygen. Competition for oxygen may explain the decrease of viable *T. delbrueckii*-214 population.

### 3.2. Chemical Analysis of Wines

Three fermentations were carried out in Narince musts containing 230 g/L sugar. Alcoholic fermentation with pure *S. cerevisiae*-1088; mixed culture completed 13 days after inoculation, whereas pure *T. delbrueckii*-214 fermentation stopped after 14 days. The pure *T. delbrueckii*-214 culture was the only condition that did not finish the fermentation (8 g/L glucose and 14 g/L fructose left in the medium) (Table 1). *S. cerevisiae*-1088 showed the highest fermentative ability in pure culture with the production of 11.6 *v*/*v*, followed by mixed culture fermentation (11.4% *v*/*v*). Fermentation performed with pure *T. delbrueckii*-214 produced the lowest ethanol 10.1% *v*/*v*. Renault et al. [28] reported that the average value of ethanol produced by all *T. delbrueckii* strains was 9.88% *v*/*v*. The volatile acidity of Narince wines was found to be between 0.40 and 0.58 g/L acetic acid. The fermentation carried out with pure *S. cerevisiae*-1088 yielded the lower volatile acidity whereas pure *T. delbrueckii*-214 yielded the highest. The level of volatile acidity in the mixed culture fermentation is between those of *T. delbrueckii*-214 and *S. cerevisiae*-1088 fermentations. *T. delbrueckii* is known for its ability to produce a low amount of acetic acid [29,30]. However in this study the fermentation with pure *T. delbrueckii*-214 produced the highest amount of volatile acidity. Glycerol is a wine constituent related to yeast metabolism which contributes to the sweetness, viscosity, and smoothness of wine [31]. In Narince wines glycerol production was greater in pure *T. delbrueckii*-214. Also the mixed *T. delbrueckii*-214/*S. cerevisaie*-1088 culture resulted in increases in glycerol, in comparison with the pure *S. cerevisae*-1088 culture. Renault et al. [28] reported that *T. delbrueckii* is a low glycerol producer, but this is strain dependent. The remaining sugar content of wines was found to be less than 3 g/L in wines produced with pure *S. cerevisiae*-1088 and mixed culture and is compatible with the concentration for dry wines. But as mentioned above, *T. delbrueckii*-214 did not ferment grape juice to dryness. 

### 3.3. Volatile Composition of the Narince Wines Produced in the Pure and Mixed Culture

Table 2 shows volatile compounds of Narince wines fermented with pure and mixed culture of *T. delbrueckii*-214 and *S. cerevisiae*-1088. A total of 47 volatile compounds were quantified in Narince wines including 15 higher alcohols, 15 esters, 10 volatile acids, two carbonyl compounds, three lactones, and two volatile phenols. Significant differences in the volatile composition of the wines were observed in those obtained from mixed culture fermentations, as well as those obtained from pure culture fermentations. The pure *T. delbrueckii*-214 has the highest (396.7 mg/L) concentration of total volatile compounds due to production of a high level of acetaldehyde, whereas the pure *S. cerevisiae* fermentation has the lowest (253.6 mg/L) concentration. The concentration of total volatile compounds in the mixed culture fermentation (291.2 mg/L) was found between those of the pure *T. delbrueckii*-214 and *S. cerevisiae*-1088 fermentation.

Higher alcohols are produced from the Erlich pathway in the presence of amino acids and from sugar via biosynthesis by yeasts during alcoholic fermentation [32]. This group was the largest group of volatile compounds determined in Narince wines. The wine fermented with mixed culture had the highest total higher alcohol concentration (219.9 mg/L), followed by the wine fermented with pure *S. cerevisiae*-1088 (191.7 mg/L), and the wine fermented with pure *T. delbrueckii*-214 (186.9 mg/L). At concentrations below 300 mg/L, they contribute to the desirable complexity of wine. When their concentration exceeds 400 mg/L, higher alcohols are regarded as a negative quality factor [33,34]. In all Narince wines, the concentrations of total higher alcohols found were below the 300 mg/L threshold. Isoamyl alcohol and 2-phenyl ethanol were the most abundant higher alcohols in all Narince wines. The wine fermented with mixed culture had highest concentration of isoamyl alcohol. However, the wine fermented with pure culture of *T. delbrueckii*-214 presented a significantly higher concentration of 2-phenyl ethanol. The odor threshold values of isoamyl alcohol and 2-phenylethyl alcohol are 30 mg/L and 10 mg/L, respectively. Isoamyl alcohol has a harsh aroma, whereas 2-phenylethyl alcohol has a floral and rose-like aroma and contributes positively to wine aroma [33]. Concentration of these compounds exceeded their threshold values in all Narince wines. The development of *T. delbrueckii*-214 also significantly increased the production of certain higher alcohols, such as 1-propanol, n-butanol, 3-ethoxy-1-propanol, and methionol in wines. Methionol has cooked potato, cauliflower notes and it exceed its threshold value of 1 mg/L [10] in wine fermented with pure *T. delbrueckii*-214. Azzolini et al. reported [35] that *T. delbrueckii* increased the amount of methionol in Chardonnay wines.

Esters are an important group of volatile compounds and they are mainly produced by yeast metabolism during alcoholic fermentation [33]. Commercial wine strains produce different amounts of esters, such as ethyl acetate, isoamyl acetate, hexyl acetate, ethyl hexanoate, and ethyl octanoate, which have a potential influence on the aroma profile of wine. Also, there are several non-*Saccharomyces* wine yeasts that can positively effect of the ester profiles of wines which can be used as a mixed culture with *S. cerevisiae* [4,33]. The pure *S. cerevisiae*-1088 fermentation produced less total esters (41.4 mg/L) than the others. In the mixed culture fermentation, overall ester production was higher (49.2 mg/L) than in both pure cultures. The concentration of total esters in the pure *T. delbrueckii*-214 fermentation (45.5 mg/L) was found between those of the pure *S. cerevisiae*-1088 and mixed culture fermentations. It has been reported that ester production by *T. delbrueckii* is strain-dependent and results are different when *T. delbrueckii* is associated with *S. cerevisiae* in mixed culture [1]. Ethyl acetate was found as the highest amount of ester compound in all Narince wines. This compound may contribute with a pleasant fruity fragrance to the general wine aroma at low concentration, whereas it contributes significantly do defect aroma at a concentration between 150 and 200 mg/L [34]. In the present study, the concentration of ethyl acetate was found between 29.2 mg/L and 38.7 mg/L and the pure *T. delbrueckii*-214 produced the highest amount of ethyl acetate in Narince wine. The aroma threshold value of ethyl acetate is 7.5 mg/L [33], and it exceeded its odor threshold value in all Narince wines. Other esters, isoamyl acetate, ethyl hexanoate, hexyl acetate, ethyl octanoate, ethyl decanoate, diethyl succinate, and ethyl-4-hydroxybutyrate were found at much lower concentrations in the pure *T. delbrueckii*-214 fermentation compared to the pure *S. cerevisiae*-1088 and mixed culture fermentations. On the other hand, ethyl lactate and ethyl hydrogen succinate were found at higher concentration in the pure *T. delbrueckii*-214 fermentation. Nevertheless, the concentrations of isoamyl acetate (0.03 mg/L), ethyl hexanoate (0.05 mg/L), and ethyl octanoate (0.02 mg/L) were detected at levels much higher than their threshold values [17] in all Narince wines. These compounds give wine pleasant aromas such as banana, pear, green apple, sweet soap, respectively [33].

The production of volatile acids in Narince wines by pure inoculation with *S. cerevisiae*-1088 was found to be significantly higher (8.2 mg/L). *T. delbrueckii*-214 fermentation produced much lover (3.9 mg/L) volatile acids than the others. But *T. delbrueckii*-214 significantly affected the accumulation of isobutyric acid and 9-decanoic acid. As indicated in Table 2, isobutyric acid was the most abundant volatile acid in wine fermented with pure *T. delbrueckii*-214, whereas hexanoic and octanoic acids were the most abundant volatile acids in wines fermented with pure *S. cerevisiae*-1088 and mixed culture.

Two carbonyl compounds, acetoin and acetaldehyde were detected in all Narince wines. Acetaldehyde is a major carbonyl compound found in wine with concentration 10 mg/L–75 mg/L and the sensory threshold value 100 mg/L [17,32]. At low levels it gives pleasant fruity aroma, but at high concentrations it has a pungent irritating odor [36]. In Narince wines acetaldehyde concentration was found between 9.6 mg/L and 157.0 mg/L. Acetaldehyde concentration was found to be higher in wine fermented with pure *T. delbrueckii*-214 culture and it exceeded its threshold value. Acetaldehyde production by yeasts effected by various factors, such as temperature, oxygen, and SO_2_ [36] *T. delbrueckii* species is considered to have a high industrial potential due to its low production of undesirable byproducts such as acetaldehyde and acetoin [11,12]. However in this study, the concentration of acetoin and acetaldehyde were found higher in wine inoculated with pure *T. delbrueckii*-214. The production of carbonyl compounds by mixed and pure *S.cerevisiae*-1088 cultures was significantly lower than in the pure *T. delbrueckii*-2014. 

Table 2 shows the lactones of the Narince wines. Total lactones were found higher in the wine inoculated with pure *S. cerevisiae*-1088. This increase was due to γ-butyrolactone. However the concentration of γ-butyrolactone found was much lower than its threshold value of 35 mg/L [33] in all Narince wines.

Two volatile phenols, 4-vinylguaciol and acetovanillone were detected in Narince wines. Both *T. delbrueckii*-214 and *S. cerevisiae*-1088 produced small amounts of volatile phenols. However, 4-vinylguaicol and acetovanillone were found higher in wine inoculated with pure *T. delbrueckii*-214 compare to wine inoculated with pure *S. cerevisae*-1088 and mixed culture. Odor threshold values of 4-vinylguaciol and acetovanillone are 10 mg/L [33] and 1 mg/L [37], respectively. But concentrations of these compounds found below their threshold values.

### 3.4. Sensory Analysis of the Narince Wines Produced in the Pure and Mixed Culture

**Figure 3:**
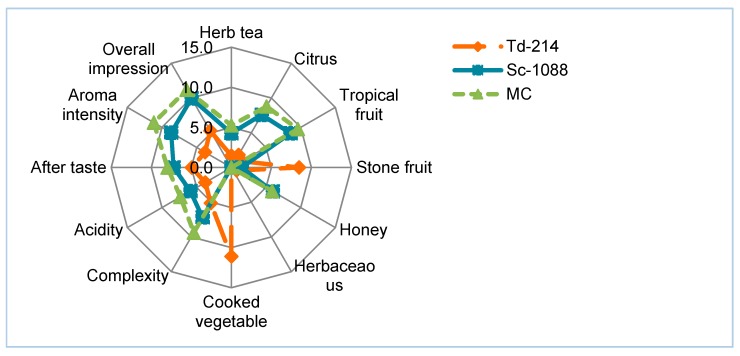
Sensory analysis of Narince wines produced in the pure and mixed culture. Td-214: *T. delbrueckii*-214, Sc-1088: *S. cerevisiae*-1088, MC: Mixed culture of *T. delbrueckii*-214 and *S.cerevisiae*-1088.

Figure 3 shows the sensory analysis results of Narince wines. Wine fermented with mixed culture of *T. delbrueckii*-214 and *S. cerevisiae*-1088 obtained higher scores in herbal tea, citrus, tropical fruit, honey attributes, and followed by the wine fermented with pure *S. cerevisiae*-1088 culture. The wine fermented with pure *T. delbrueckii*-214 scored highest in stone fruit and cooked vegetable attributes. The unpleasant cooked vegetable attribute was probably due to higher level production of methionol. The aroma intensity and complexity attributes were greater in mixed culture fermentation compared to pure culture fermentations. This Narince wine, which is fermented with mixed culture, is the most preferred wine compared to others. Gonzales-Rayo et al. [11] have reported that Macabeo wine inoculated with a mixed culture of *T. delbrueckii* and *S. cerevisiae* was the most preferred wine among the others.

## 4. Conclusions

The current study examined the effects of pure and mixed autochthonous *T. delbrueckii*-214 and *S.cerevisiae*-1088 on fermentation and volatile composition of Narince wines. Fermentation with mixed culture of *T. delbrueckii*-214/*S. cerevisiae*-1088 demonstrated significant differences distinguishing the chemical and sensory properties, compared to pure culture fermentations with *T. delbrueckii*-214 and *S. cerevisiae*-1088. Autochthonous *T. delbrueckii*-214 yeast slowed down the fermentation and produced a lower level of ethanol and a higher level of glycerol and volatile acid. *T. delbrueckii* also has some effects on wine quality and aroma composition during fermentation. These effects are a reduction of the main ester concentrations, an increase in concentration of carbonyl compounds, some higher alcohols, and minor ethyl esters. The results indicated that the presence of *T. delbrueckii*-214 in the mixed culture fermentation significantly increased the levels of isoamyl alcohol, 2-phenyl ethanol, ethyl acetate, isoamyl acetate, ethyl lactate, ethyl hydrogen succinate, and isobutanoic acid that may be responsible for, and improved the intensity of, the aroma and complexity of Narince wine. However, industrial large-scale fermentations are needed to confirm these results.

## Figures and Tables

**Table 1:** General compositions of Narince wines.

*General Composition*	Td-214	Sc-1088	MC	F
Alcohol (%*v*/*v*)	10.12 ± 0.0 ^a^	11.6 ± 0.2 ^b^	11.48 ± 0.0 ^b^	*
Total acidity **	7.04 ± 0.0 ^b^	6.05 ± 0.0 ^a^	6.56 ± 0.0 ^b^	*
pH	3.68 ± 0.0 ^a,b^	3.66 ± 0.0 ^a^	3.71 ± 0.0 ^b^	*
Volatile acidity (g/L) ***	0.58 ± 0.0 ^c^	0.40 ± 0.0 ^a^	0.43 ± 0.0 ^b^	*
Total SO_2_	56 ± 1	56 ± 0.5	57 ± 0.2	ns
Dry matter (g/L)	21.6 ± 0.0	21.9 ± 0.0	21.7 ± 0.0	ns
Glycerol	7.37 ± 0.3 ^b^	5.93 ± 0.0 ^a^	6.06 ± 0.1 ^a^	*
**Sugars (g/L)**				
Glucose	8.06 ± 0.1 ^c^	0.95 ± 0.0 ^a^	1.26 ± 0.0 ^b^	*
Fructose	14.25 ± 0.0 ^c^	0.87 ± 0.0 ^a^	1.52 ± 0.0 ^b^	*
Total	22.31	1.82	2.78	

Td-214: *T. delbrueckii*-214, Sc-1088: *S. cerevisiae*-1088, MC: Mixed culture of *T. delbrueckii*-2014 and *S. cerevisiae*-1088. F: significance at which means differ as shown using analysis of variance * *p* < 0.05 level. **: As tartaric acid, ***: As acetic acid.

**Table 2:** Volatile composition of the Narince wines produced in the pure and mixed culture.

AROMAC OMPOUNDS (µg/L)						
***Higher alcohols***	**RI**	**Td-214**	**Sc-1088**	**Td + Sc**	**F**	**ID**
1-Propanol	1037	1702.4 ± 7 ^b^	1224.5 ± 64 ^a^	1283.0 ± 5 ^a^	*	RI, MS, Std
Isobutyl alcohol	1085	10,621.5 ± 62 ^b^	9123.2 ± 153 ^a^	10,649.2 ± 1012 ^b^	*	RI, MS, Std
1-Butanol	1165	617.9 ± 1 ^c^	309.6 ± 29 ^a^	400.5 ± 59 ^b^	*	RI, MS, Std
Isoamyl alcohol	1210	120,544.6 ± 757 ^a^	144,208.5 ± 1146 ^b^	167,995.9 ± 459 ^c^	*	RI, MS, Std
2-Hexanol	1226	200.0 ± 9 ^a^	295.0 ± 20 ^c^	246.4 ± 13 ^b^	*	RI, MS, Std
3-Pentanol	1295	104.0 ± 2 ^c^	67.2 ± 0 ^a^	73.6 ± 1 ^b^	*	RI, MS, Std
1-Hexanol	1370	1329.5 ± 4 ^b^	1249.5 ± 25 ^a^	1228.9 ± 18 ^a^	*	RI, MS, Std
(E)-3-Hexen-1-ol	1386	3.2 ± 0 ^a^	136.8 ± 4 ^c^	116.4 ± 2 ^b^	*	RI, MS, Std
3-Ethoxy-1-propanol	1390	741.6 ± 1 ^c^	43.9 ± 1 ^a^	162.9 ± 5 ^b^	*	RI, MS, Std
(Z)-3-Hexen-1-nol	1401	104.4 ± 4	103.2 ± 5	93.4 ± 7	ns	RI, MS, Std
2,3-Butanediol	1495	771.6 ± 9 ^a^	1371.7 ± 19 ^c^	1029.1 ± 42 ^b^	*	RI, MS, Std
1,3-Butanediol	1566	296.5 ± 2 ^c^	239.7 ± 1 ^b^	215.9 ± 13 ^a^	*	RI, MS
Methionole	1737	1746.5 ± 8 ^c^	590.9 ± 5 ^a^	722.7 ± 11 ^b^	*	RI, MS, Std
Benzyl alcohol	1804	36.2 ± 2 ^a^	59.9 ± 4 ^c^	46.9 ± 0 ^b^	*	RI, MS, Std
2-Phenyl ethanol	1916	48,153.0 ± 337 ^c^	32,738.2 ± 584 ^a^	35,718.7 ± 765 ^b^	*	RI, MS, Std
***Sum***		**186,971.5**	**191,755.8**	**219,983.5**		
***Esters***						
Ethyl acetate **	895	38,712.4 ± 1266 ^b^	29,309.0 ± 499 ^a^	36,891.9 ± 1894 ^b^	*	MS, RI
Isoamyl acetate	1119	646.6 ± 0 ^a^	1336.7 ± 37 ^b^	1565.2 ± 62 ^c^	*	RI, MS, Std
Ethyl hexanoate	1241	826.5 ± 8 ^a^	1403.6 ± 6 ^b^	1438.5 ± 41 ^b^	*	RI, MS, Std
Hexyl acetate	1250	117.9 ± 0 ^a^	202.6 ± 7 ^b^	190.7 ± 12 ^b^	*	RI, MS, Std
Ethyl lactate	1353	978.2 ± 4 ^c^	609.0 ± 8 ^a^	775.6 ± 18 ^b^	*	RI, MS, Std
Ethyl octanoate	1430	173.8 ± 5 ^a^	1226.1 ± 8 ^c^	1052.6 ± 10 ^b^	*	RI, MS, Std
Ethyl-3-hydroxybutyrate	1524	nd	109.2 ± 1 ^a^	116.4 ± 2 ^b^	*	RI, MS, Std
Ethyl decanoate	1635	28.3 ± 2 ^a^	300.3 ± 1 ^c^	232.2 ± 7 ^b^	*	RI, MS, Std
1,3-Propanediol diacetate	1650	258.1 ± 0 ^c^	194.5 ± 7 ^a^	223.8 ± 6 ^b^	*	RI, MS
Diethyl succinate	1690	152.2 ± 4 ^a^	248.9 ± 5 ^c^	215.9 ± 4 ^b^	*	RI, MS, Std
Ethyl-4-hydroxybutyrate	1819	656.6 ± 0 ^a^	4202.7 ± 22 ^c^	3945.0 ± 120 ^b^	*	RI, MS
Ethyl dodecanoate	1851	26.6 ± 1 ^a^	83.2 ± 3 ^c^	73.6 ± 0 ^b^	*	RI, MS, Std
Diethyl-dL-malate	2041	108.3 ± 10	115.2 ± 2	116.8 ± 1	ns	RI, MS, Std
Ethyl hexadecanoate	2259	132.9 ± 2 ^a^	234.1 ± 12 ^b^	124.4 ± 6 ^a^	*	RI, MS, Std
Ethyl hydrogen succinate	2331	2778.7 ± 63 ^c^	1884.5 ± 106 ^a^	2524.4 ± 169 ^b^	*	RI, MS
***Sum***		**45,597.1**	**41,459.6**	**49,263.2**		
***Volatile acids***						
Propionic acid	1538	53.5 ± 0 ^a^	71.8 ± 0 ^b^	71.7 ± 2 ^b^	*	RI, MS, Std
Isobutanoic acid	1584	1052.1 ± 6 ^c^	373.1 ± 5 ^a^	526.5 ± 23 ^b^	*	RI, MS, Std
Butanoic acid	1628	211.6 ± 4 ^a^	295.5 ± 4 ^b^	338.4 ± 15 ^c^	*	RI, MS, Std
Isovaleric acid	1608	365.1 ± 5 ^a^	633.0 ± 11 ^b^	625.8 ± 14 ^b^	*	RI, MS, Std
Hexanoic acid	1840	787.6 ± 3 ^a^	2355.7 ± 70 ^b^	2381.1 ± 62 ^b^	*	RI, MS, Std
(E)-2-Hexenoic acid	1962	150.0 ± 13	134.9 ± 6	128.3 ± 6	ns	RI, MS, Std
Octanoic acid	2060	624.6 ± 3 ^a^	3383.2 ± 112 ^c^	3152.5 ± 98 ^b^	*	RI, MS, Std
Nonanoic acid	2158	94.1 ± 1	101.5 ± 26	70.6 ± 6	ns	RI, MS, Std
Decanoic acid	2183	40.6 ± 1 ^a^	573.1 ± 36 ^b^	623.3 ± 16 ^c^	*	RI, MS
9-Decenoic acid	2237	611.4 ± 11 ^b^	293.9 ± 17 ^a^	269.5 ± 9 ^a^	*	RI, MS
***Sum***		**3990.6**	**8215.6**	**8187.9**		
***Carbonyl compounds***	**RI**	**Td-214**	**Sc-1088**	**MC**	**F**	**ID**
Acetoin	1291	1448.5 ± 3 ^b^	307.9 ± 4 ^a^	299.1 ± 21 ^a^	*	RI, MS, Std
Acetaldehyde **	500	157,040.7 ± 3248 ^b^	9631.6 ± 499 ^a^	11,237.0 ± 726 ^a^	*	MS, RI
***Sum***		**158,489.2**	**9939.5**	**11,536.1**		
***Lactones***						
γ-Butyrolactone	1635	1185.2 ± 4 ^a^	1726.8 ± 12 ^b^	1770.8 ± 46 ^b^	*	RI, MS, Std
Pantolactone	2414	146.7 ± 3 ^b^	83.9 ± 22 ^a^	59.6 ± 1 ^a^	*	RI, MS, Std
4-Ethoxycarbonyl-γ-butyrolactone	2673	85.9 ± 1 ^a^	262.4 ± 7 ^c^	229.9 ± 0 ^b^	*	RI, MS
***Sum***		**1417.9**	**2073**	**2060.4**		
***Volatile phenols***						
4-Vinylguaicol	2091	215.3 ± 3 ^c^	198.6 ± 0 ^b^	172.4 ± 6 ^a^	*	RI, MS, Std
Acetovanillone	2995	50.3 ± 0 ^b^	54.6 ± 0 ^c^	32.2 ± 0 ^a^	*	RI, MS, Std
***Sum***		**265.6**	**253.2**	**204.6**		
**Total Sum**		**396,731.9**	**253,696.7**	**291,235.7**		

Td-214: *T. delbrueckii*-214, Sc-1088: *S. cerevisiae*-1088, MC: Mixed culture of *T. delbrueckii*-2014 and *S. cerevisiae*-1088. RI: retention index calculated on DBWax capillary column, MS: mass spectrometry, Std: chemical standard, ±: Standard deviation, nd: not detected, F: significance at which means differ as shown using analysis of variance, *: important at *p* < 0.05 level, a-c: different superscripts in the same row indicate statistical differences at the *p* < 0.05 level, ns: not significant. **: Determined by direct injection into GC.
